# Acute Aerobic Exercise in Individuals with Obesity Abolishes Amino Acid-Stimulated Muscle Protein Synthesis in the Immediate Postexercise Period

**DOI:** 10.64898/2026.06.14.732200

**Published:** 2026-06-18

**Authors:** Kailin A. Johnson, Eduardo D. S. Freitas, Lori R. Roust, Eleanna De Filippis, Haiwei Gu, Matthew Buras, Christos S. Katsanos

**Affiliations:** 1 School of Life Sciences, Arizona State University, Tempe, AZ 85259; 2 College of Medicine, Mayo Clinic Arizona, Scottsdale, AZ 85259; 3 College of Health Solutions, Arizona State University, Phoenix, AZ 85004; 4 Department of Quantitative Health Sciences, Mayo Clinic Arizona, Scottsdale, AZ 85259; 5 Department of Physiology and Biomedical Engineering, Mayo Clinic Arizona, Scottsdale, AZ 85259

**Keywords:** obesity, aerobic exercise, amino acids, muscle protein synthesis, anabolic resistance

## Abstract

Obesity alters protein metabolism in skeletal muscle, and although exercise and amino acids act synergistically to regulate muscle anabolism in healthy humans, this interaction may be impaired in obesity. We examined whether acute aerobic exercise alters amino acid–stimulated muscle protein synthesis during the immediate postexercise period in subjects with obesity. Sixteen sedentary adults with a body mass index >30 kg/m^2^ underwent stable-isotope tracer infusion studies to determine mixed-muscle fractional synthesis rate (FSR) in the basal (fasted) state and under two experimental conditions: eight subjects received an amino acid infusion (AA), while another eight performed 45 min of cycling at ~65% heart rate reserve immediately prior to the amino acid infusion (EX+AA). Amino acid infusion significantly increased muscle protein FSR in AA (*P* < 0.0001). In contrast, no significant increase was observed in EX+AA (*P* > 0.05), and the amino acid-stimulated increase in muscle protein FSR in EX+AA was 78% lower than that in the AA (*P* < 0.01). Amino acid infusion increased plasma amino acid concentrations in both conditions (*P* < 0.05); however, plasma concentrations of essential and branched-chain amino acids, including leucine, were lower in the EX+AA condition (*P* < 0.05). Changes in muscle protein FSR were positively associated with plasma leucine concentrations during the amino acid infusion (*P* < 0.05). These findings suggest that, in humans with obesity, aerobic exercise may abolish amino acid-stimulated muscle protein synthesis during the immediate postexercise period, with implications when considering nutritional strategies designed to optimize muscle anabolism in this population.

## Graphical Abstract

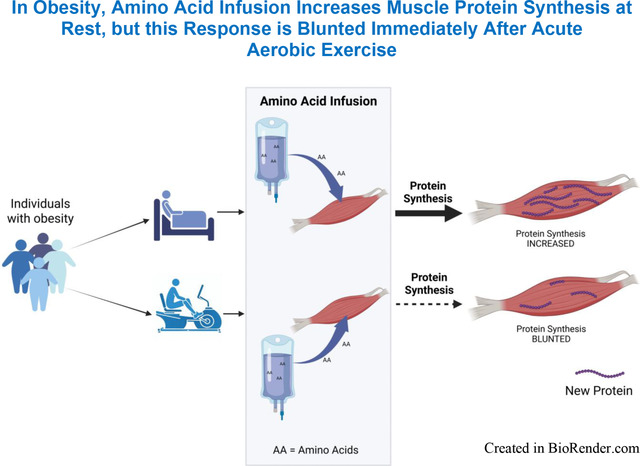

## INTRODUCTION

Although obesity has traditionally been viewed primarily as a disorder characterized by impairments in skeletal muscle glucose and lipid metabolism (1, 2), growing evidence now indicates that obesity also negatively impacts muscle protein metabolism (3, 4). Circulating amino acids serve as principal anabolic stimuli for muscle protein synthesis (5, 6), and attenuated anabolic responses to experimental elevations in plasma amino acid concentrations have been reported in individuals with obesity (7), although this effect is not consistent across studies (8).

It has long been recognized that exercise is a potent regulator of skeletal muscle protein metabolism, and that the postexercise metabolic milieu and combination of increased plasma amino acid availability maximizes the stimulation of muscle protein synthesis (9, 10). This effect has been studied primarily in the context of resistance exercise, where prior exercise potentiates the anabolic responsiveness of skeletal muscle to elevations in plasma amino acids (11). The interaction between exercise and nutrient provision is considered a fundamental determinant of skeletal muscle adaptation, and forms the physiological basis for recommendations advocating the coupling of exercise with adequate dietary protein intake (12). However, emerging evidence suggests that this synergistic interaction between exercise and amino acid availability in stimulating protein synthesis in muscle may be impaired in obesity. For example, Beals et al. (13) demonstrated that obesity attenuates the capacity of prior resistance exercise to enhance fed-state protein synthesis in muscle.

While resistance exercise has received the greatest attention in the context of nutrient-stimulated muscle protein synthesis, aerobic exercise also has the capacity to stimulate significant increases in protein synthesis in muscle (14), albeit to a lesser magnitude than resistance exercise performed immediately prior to nutrient provision (15). Understanding the interaction between aerobic exercise and amino acid availability in regulating muscle protein synthesis in individuals with obesity is of particular importance, as aerobic exercise remains one of the most commonly prescribed and widely adopted interventions for obesity management.

While aerobic exercise does not appear to alter the rate of muscle protein synthesis during the exercise bout itself (16), protein synthesis increases during the postexercise recovery period in healthy adults (14, 17). In the postabsorptive state in healthy humans, although findings are not entirely consistent (18), aerobic exercise has been reported to increase mixed-muscle protein synthesis rates by approximately 45% (16), and comparable findings have been reported subsequently (19). However, the immediate post-exercise period is also characterized by a transient, exercise-induced cellular stress response in muscle (20, 21), and obesity may exacerbate this response (22), potentially impairing the postexercise increase in muscle protein synthesis. As such, it is plausible that acute aerobic exercise in individuals with obesity could transiently diminish the muscle anabolic responsiveness to increased plasma amino acid availability during the immediate post-exercise period, despite evidence that hyperaminoacidemia alone effectively stimulates muscle protein synthesis (5), including in humans with obesity (8). Accordingly, we determined whether acute aerobic exercise alters the amino acid-stimulated muscle protein synthesis during the immediate postexercise period in individuals with obesity.

## METHODS

### Study Participants

We studied sixteen individuals with obesity (body mass index [BMI] >30 kg/m^2^) who did not engage in regular physical activity or exercise more than two days per week and did not meet current physical activity recommendations (23). All study procedures were approved by the Institutional Review Board at Mayo Clinic, and written informed consent was obtained from each participant. Prior to obtaining written consent, the study purpose, requirements, and potential risks associated with study participation were explained to each participant.

Participants first underwent screening within the Ambulatory Infusion Unit (AIC) at Mayo Clinic in Arizona. Eligibility was determined based on medical history, physical examination, electrocardiogram, and standard blood and urine analyses, confirming that all participants were otherwise healthy. Exclusion criteria included the presence of acute illness, diabetes, liver, renal, or cardiovascular disease, and chronically elevated blood pressure (systolic, >150 mmHg; diastolic, >100 mmHg). Participants were also excluded if they were actively engaged in a weight-loss program, smoked, or used nutritional supplements, prescription medications, or over-the-counter drugs. During the screening visit, insulin sensitivity was estimated using the Matsuda-Insulin Sensitivity Index (ISI) (24). The Matsuda-ISI was calculated from plasma insulin and glucose responses obtained during an oral glucose tolerance test (OGTT), as previously described (24). Following the OGTT, participants were included only if fasting plasma glucose was <126 mg/dL and 2-h plasma glucose was <200 mg/dL, consistent with the absence of diabetes.

Participants returned to the AIC on a separate day for assessment of body composition using dual-energy X-ray absorptiometry (Hologic, Inc). This assessment was followed by the determination of peak oxygen consumption (VO_2_peak) using an incremental cycle ergometer test. For this test, after a 5-min unloaded warm-up, work rate was increased by 20 W·min^−1^ while participants were instructed to maintain a pedaling cadence of 65 revolutions·min^−1^ until volitional exhaustion. During the VO_2_peak test, a 12-lead electrocardiogram was continuously recorded, and blood pressure and oxygen saturation were monitored throughout the test. Participants were verbally encouraged to exercise until exhaustion (25), and all achieved a respiratory exchange ratio >1.1 prior to test termination, consistent with maximal effort (26). Expired gases were continuously analyzed using a metabolic cart (MedGraphics, Saint Paul, MN).

### Experimental Design

On a separate day, participants arrived at the AIC at ~7:00 AM after a 10–12 h overnight fast to undergo a stable-isotope tracer infusion study to measure skeletal muscle protein synthesis rates before (Basal) and during an intravenous amino acid infusion (AA) designed to increase plasma amino acid concentrations and stimulate protein synthesis in muscle. Participants assigned to the exercise condition performed a single session of aerobic exercise following the basal measurements and immediately prior to the initiation of the amino acid infusion (EX+AA). The AA condition served as control. All participants were instructed to refrain from structured exercise, maintain their habitual diet, and avoid alcohol consumption for the 3 days preceding each study visit. Adherence to fasting and physical activity instructions was confirmed prior to initiation of the tracer infusion study.

While resting in bed, participants had one catheter placed into an antecubital arm vein for infusion of d_10_ leucine (L-[2,3,3,4,5,5,5,6,6,6-^2^H_10_]leucine), initiated at time 0 at a rate of 0.15 μmolηkg FFM^−1^ηmin^−1^ (priming dose, 6.4 μmolηkg FFM^−1^), and maintained throughout the study to assess mixed-muscle protein synthesis rates. A separate catheter was placed retrogradely in a dorsal hand vein for blood sampling using the heated-hand technique. Following sample collection at the end of the Basal period (0–270 min), a primed continuous infusion of an amino acid solution (15% Clinisol, Baxter Healthcare Corporation, Deerfield, IL) was initiated at 4 mgηkg FFM^−1^ηmin^−1^ (priming dose, 82 mgηkg FFM^−1^) and continued for 240 mins (i.e., Amino Acid Infusion period). During the amino acid infusion, the infusion rate of d_10_-leucine was increased to 0.29 μmolηkg FFM^−1^ηmin^−1^ (priming dose, 2.6 μmolηkg FFM^−1^) to account for tracer dilution resulting from the exogenous (i.e., infused) leucine delivery.

For the EX+AA condition, participants performed 45 min of cycling exercise following completion of the basal measurements and prior to the initiation of the amino acid infusion, which was administered for the same duration (240 min) as in the AA condition of the study. The exercise was performed at an intensity corresponding to 65% of heart rate reserve, calculated from each participant’s resting heart rate and the maximal heart rate achieved during the VO_2_peak test (27). During the exercise session, the workload on the cycle ergometer was adjusted as needed to maintain heart rate within ±5 bpm of the target exercise intensity. Following completion of the exercise session, participants rested in bed for the remainder of the infusion protocol.

Two muscle biopsy samples (~70 mg) were collected from the vastus lateralis muscle at 120 and 270 min (biopsies 1 and 2, respectively) following the start of the d_10_-leucine infusion (i.e., Basal), and an additional biopsy (biopsy 3) was collected at the end of the 240-min amino acid infusion period. Muscle tissue samples were immediately cleaned of visible fat and connective tissue, blotted dry, and frozen in liquid nitrogen. Blood samples were collected for the determination of d_9_-leucine enrichment (28) and selected biochemical parameters during the Basal and Amino Acid Infusion periods. Blood samples were collected at the following time points during the Basal period: 0, 110, 250, and 260 min; during the Amino Acid Infusion period, samples were collected at 30, 40, 55, 70, 90, 110, 210, and 220 min after initiation of the amino acid infusion. We have previously validated the use of plasma d_9_-leucine enrichment measured at selected time points to quantify skeletal muscle protein fractional synthesis rate (FSR) (28). Blood samples collected at these time points were also used to measure relevant biochemical variables in the course of the infusion study.

### Sample Analyses

Leucine enrichment (i.e., d_9_-leucine) was measured in blood and mixed-muscle protein using procedures we have previously described (8, 28). Briefly, proteins in 1 mL of blood were precipitated using sulfosalicylic acid (SSA). Mixed-muscle samples (~15 mg) were similarly processed by homogenization in SSA to precipitate proteins. Proteins in both blood and mixed-muscle samples were subsequently hydrolyzed with 6 N HCl. Amino acids were then isolated using cation-exchange chromatography, and d_9_-leucine enrichment was determined by liquid chromatography-tandem mass spectrometry (LC-MS/MS) at the Mayo Clinic Metabolomics Core. Plasma d_9_-leucine enrichment was measured during the Basal period at 0, 110, 250, and 260 min time points, and at 30, 40, 210, and 220 min during the Amino Acid Infusion period. Mixed-muscle protein enrichment was determined at each muscle biopsy and used to calculate the mixed-muscle protein fractional synthesis rate (FSR; %·h^−1^) during the Basal period, between biopsies 1 and 2, and for the Amino Acid Infusion period, between biopsies 2 and 3, using the precursor-product approach, as we have previously described (28).

Plasma amino acid concentrations were measured by LC-MS/MS using a method adapted from previously published protocols (29–31). Analyses were performed using an Agilent 1290 UPLC–6495 QQQ-MS (Santa Clara, CA) system, with chromatographic separation conducted under hydrophilic interaction chromatography conditions on a Waters XBridge BEH Amide column (Waters Corporation, Milford, MA). The mass spectrometer was operated with an electrospray ionization source, and amino acids were quantified using targeted multiple-reaction monitoring. Peak integration was performed with Agilent MassHunter Quantitative Data Analysis software (Santa Clara, CA). Individual plasma amino acid concentrations were quantified at 260 min during the Basal period and at 55 and 210 mins during the Amino Acid Infusion Period. Plasma amino acid concentrations were also summarized as the sum of measured total amino acids (TAA), essential amino acids (EAA), branched-chain amino acids (BCAA), and non-essential amino acids (NEAA), given the central role of EAA, and particularly BCAA, in stimulating skeletal muscle protein synthesis (5, 32, 33).

Plasma glucose and insulin concentrations were measured at 0 and 260 min during the Basal period and at 40, 55, 70, 90, 110, and 210 min during the Amino Acid Infusion Period. Plasma glucose concentrations were determined using an automated glucose analyzer (YSI 2300, Yellow Springs, OH). Plasma insulin concentrations were measured using a commercially available enzyme-linked immunosorbent assay kit (80-INSHU-E01.1; ALPCO Diagnostics). Clinical chemistry parameters assessed during the screening visit were analyzed at the Mayo Clinic Laboratories (Mayo Clinic, Arizona).

### Statistical Analyses

Participant characteristics and within-condition changes from Basal were compared using unpaired and paired Student’s *t*-tests, respectively. Two-way repeated-measures ANOVA (condition × amino acid infusion) was used to assess the main effects of condition and amino acid infusion on variables of interest. Pairwise comparisons were conducted using the Bonferroni correction. Associations between variables of interest were evaluated using Pearson’s product-moment correlation coefficient (*r*). Data are presented as mean ± SEM, and results with *P* < 0.05 were considered statistically significant. All statistical analyses were performed using GraphPad Prism (Version 10; GraphPad Software, La Jolla, CA).

## RESULTS

Anthropometric characteristics and fasted-state blood chemistry parameters were comparable between groups, with no statistically significant differences observed for any of the measured parameters (Table 1). In addition to being classified as physically inactive during screening, as described in the Methods, participants in both groups exhibited a VO_2_peak of ~22 ml·kg^−1^·min^−1^, consistent with low cardiorespiratory fitness according to established population reference standards (34).

### Muscle Protein Synthesis

**Figure 1.**
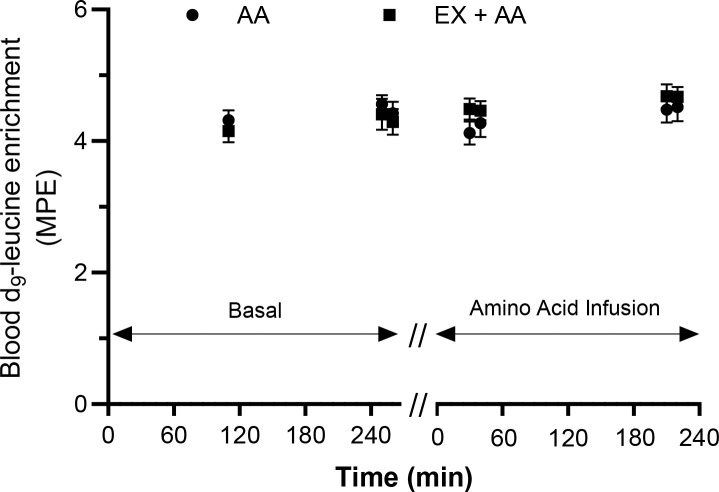
Blood d_9_-leucine enrichment. Blood d_9_-leucine enrichment during the Basal and Amino Acid Infusion periods in the amino acid infusion-only condition (AA) and the exercise plus amino acid infusion condition (EX+AA). Values are presented as means ± SEM. The interrupted x-axis denotes the separation between the Basal and Amino Acid Infusion periods.

**Figure 2.**
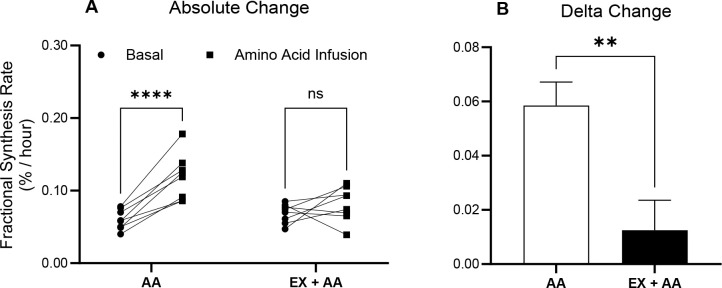
Fractional synthesis rate of muscle protein. Fractional synthesis rate was measured during the Basal and Amino Acid Infusion periods in the amino acid infusion-only condition (AA) and the exercise plus amino acid infusion condition (EX+AA). (A) Individual responses during the Basal and Amino Acid Infusion periods in the AA and EX+AA conditions. (B) Delta change in fractional synthesis rate from Basal to Amino Acid Infusion in the AA and EX+AA conditions; values are presented as means ± SEM. ****, *P* < 0.0001; **, *P* < 0.01; ns, not statistically significant.

Muscle protein synthesis, expressed as FSR, was the primary end-point of the study. Blood d_9_-leucine enrichment responses during the Basal and the Amino Acid Infusion periods in both conditions are shown in Figure 1. No significant main effects of either condition or amino acid infusion (i.e., time) were observed for plasma d_9_-leucine enrichment (*P* > 0.05). Basal muscle protein FSR did not differ between conditions (*P* > 0.05). Two-way ANOVA showed a significant main effect of amino acid infusion (*P* < 0.05) but not condition on muscle protein synthesis rate. Pairwise comparisons revealed that amino acid infusion significantly increased muscle protein synthesis compared with Basal in the AA condition (*P* < 0.0001), whereas no significant change was observed in the EX+AA condition (*P* > 0.05) (Figure 2A). Figure 2A also depicts individual responses in muscle protein synthesis to amino acid infusion in both conditions. Average delta changes in muscle protein synthesis in the two conditions are summarized in Figure 2B, showing that the increase in amino acid-stimulated muscle protein synthesis was 78% lower in the EX+AA condition compared with the AA condition (*P* < 0.01).

### Plasma Amino Acids

Table 2 shows plasma concentrations of individual amino acids, as well as TAA, EAA, BCAA, and NEAA, during the Basal and the Amino Acid Infusion periods. ANOVA revealed a significant main effect of amino acid infusion (time; *P* < 0.05) in the concentration for all measured plasma amino acids, with the exception of tyrosine and asparagine, as well as for all grouped amino acid categories. Significant main effects of condition (*P* < 0.05) were observed only for plasma arginine, leucine, and phenylalanine concentrations. Table 2 also shows the corresponding delta changes from Basal in the plasma amino acid concentrations during the amino acid infusion within the AA and EX+AA conditions. These responses differed between conditions (*P* < 0.05) for plasma alanine, arginine, leucine, methionine, phenylalanine, serine, threonine, and valine. Specifically, the increase was lower in the EX+AA condition for plasma alanine, leucine, methionine, phenylalanine, serine, and valine, but higher for arginine and threonine concentrations. Accordingly, the increases in plasma TAA, EAA, and BCAA, but not NEAA, concentrations were significantly lower (*P* < 0.05) in the EX+AA condition compared with the AA condition during the amino acid infusion.

Exploratory correlation analyses demonstrated that changes in plasma amino acid-stimulated muscle protein FSR from Basal were significantly correlated with circulating concentrations of specific amino acids during the amino acid infusion. Specifically, the delta change in muscle protein FSR was inversely correlated with plasma arginine (*r* = −0.62, *P* = 0.01) and glycine (*r* = −0.50, *P* = 0.04) concentrations, and positively correlated with plasma leucine concentration (*r* = 0.62, *P* = 0.01) measured in the Amino Acid Infusion period. In contrast, no significant correlations were observed for the other branched-chain amino acids, isoleucine (*P* = 0.93) and valine (*P* = 0.43). Furthermore, the change in muscle protein FSR was not significantly associated with the plasma concentrations of TAA (*P* = 0.97), EAA (*P* = 0.28), BCAA (*P* = 0.07), or NEAA (*P* = 0.15) during the amino acid infusion.

### Plasma Glucose and Insulin

Table 3 shows plasma glucose and insulin concentrations averaged across measurements obtained during the Basal and Amino Acid Infusion periods, together with the corresponding delta changes from Basal values. ANOVA revealed a significant main effect of condition (*P* < 0.01), but not time, for plasma glucose concentrations, whereas a significant main effect of time (*P* < 0.01), but not condition, was observed for plasma insulin concentrations. Amino acid infusion significantly increased plasma insulin concentrations in both the AA and EX+AA conditions (Table 3). During the amino acid infusion, the delta changes from Basal in plasma glucose and insulin concentrations were 83% and 34% lower, respectively, in the EX+AA condition than in the AA condition. However, these changes did not differ significantly between conditions (*P* > 0.05).

## DISCUSSION

The primary finding of the present study is that, in humans with obesity, acute aerobic exercise abolished the amino acid-induced increase in mixed-muscle protein synthesis during the immediate postexercise period, suggesting impaired integration of exercise- and nutrient-derived anabolic stimuli at this time. Importantly, these findings extend beyond a simple attenuation of muscle protein synthesis in obesity. The absence of a statistically significant increase in muscle protein synthesis above Basal levels, combined with an ~80% reduction in the anabolic response compared with the condition without exercise, indicates a substantial impairment in metabolic flexibility in response to a potent anabolic stimulus in the immediate period following aerobic exercise in individuals with obesity.

An increase in plasma amino acid availability alone constitutes a potent stimulus for muscle protein synthesis (5), and has been shown to also stimulate muscle protein synthesis in humans with obesity (8, 35, 36). However, obesity is not uniformly associated with normal anabolic responsiveness across all physiological conditions, and prior work has demonstrated that it alters the sensitivity of skeletal muscle protein synthesis to anabolic stimuli such as insulin, amino acids, and feeding (7, 37). Relevant to the present findings, the stimulation of muscle protein synthesis in the immediate post-resistance exercise period is attenuated in individuals with obesity (13). The present study extends these observations by demonstrating that this impairment is more pronounced in response to aerobic exercise, as the prior work showed a reduced, but still significant, increase in protein synthesis following resistance exercise (13), whereas in the current study, no significant increase in muscle protein synthesis above Basal levels was observed following aerobic exercise (Figure 2A).

In lean, healthy individuals, the combination of aerobic exercise and increased amino acid availability is considered to act synergistically to stimulate muscle protein anabolism both during and in the immediate postexercise period (38–40). Collectively, such studies have established the concept that aerobic exercise sensitizes skeletal muscle to amino acid availability (40). The present study is the first to evaluate this interaction specifically in humans with obesity, a population of direct clinical relevance, given that aerobic exercise is commonly prescribed to improve energy expenditure, glycemic control, and cardiometabolic health in these individuals. Our findings indicate not that amino acids are ineffective in obesity, but rather that prior aerobic exercise abolishes the amino acid-induced stimulation of muscle protein synthesis during the immediate postexercise period, a response that is evident in lean, healthy individuals (38–40).

One plausible mechanism for this effect is the persistence of exercise-induced energetic stress during the early postexercise period. Acute aerobic exercise increases ATP demand and activates AMP-activated protein kinase (AMPK) (41), a key cellular energy sensor that negatively regulates skeletal muscle protein synthesis (42). Increased AMPK activation and its inhibitory effects on muscle protein synthesis have been observed during the early (e.g., ~1 h postexercise), but not later, phases of recovery following resistance exercise in humans (43). However, the magnitude of AMPK activation is dependent on the mode of exercise, with greater activation observed following aerobic compared with resistance exercise (44, 45). Moreover, this response is amplified and appears to be more prolonged in exercise-untrained compared with trained individuals (46). Thus, in sedentary, exercise-naïve individuals with obesity, such as those studied here, the aerobic exercise bout likely imposed a relatively greater energetic stress, leading to sustained activation of energy-sensing pathways and delayed restoration of anabolic sensitivity to amino acids. Collectively, these findings suggest that, in humans with obesity, aerobic exercise may shift or delay the temporal window of skeletal muscle anabolic responsiveness to increased plasma amino acid availability.

Alterations in plasma amino acid availability may also contribute to the lack of stimulation of muscle protein synthesis in response to amino acid infusion following exercise, as the exercise condition was characterized by differential changes in the plasma concentrations of specific amino acids, including reduced leucine levels. These reductions in plasma amino acids are unlikely to be attributable to differences in infusion rates, as infusion rates were normalized to fat-free mass, a method that results in comparable plasma amino acid concentrations under non-exercise conditions (7, 8). Thus, the observed changes in plasma amino acid concentrations suggest that prior aerobic exercise altered amino acid metabolism. With specific regard to leucine, the lower plasma concentration observed following exercise is consistent with evidence that aerobic exercise increases leucine oxidation rates in humans (47), including individuals with obesity (48), a response associated with reduced circulating leucine concentrations (49). Importantly, even modest reductions in plasma leucine availability can have disproportionately large effects on the stimulation of muscle protein synthesis, as previously demonstrated in the context of aging (50, 51). Also, mechanistically, reduced circulating leucine levels can impair activation of key components of the translational machinery, thereby limiting the anabolic response in skeletal muscle after exercise (52). The direct correlation we observed between prevailing plasma leucine concentrations during amino acid infusion and delta changes in muscle protein FSR across subjects suggests that lower plasma leucine availability may explain, at least in part, the reduced protein synthesis response in the EX+AA condition. Therefore, it is plausible that the lower leucine plasma availability observed in the EX+AA condition in the present study contributed, alongside other factors such as AMPK activation discussed above, to the absence of amino acid-stimulated muscle protein synthesis immediately after the exercise.

From a practical perspective, these findings have important implications for the design of exercise and nutritional strategies in obesity. The potential contribution of reduced circulating amino acid availability in the exercise condition to the observed responses suggests that interventions aimed at increasing postexercise plasma amino acid concentrations may partially mitigate this effect in individuals with obesity. However, the amino acid infusion protocol employed in the present study produced large and sustained elevations in plasma amino acid concentrations that exceed those typically achieved under physiological conditions, making it unlikely that simply increasing amino acid availability, such as through dietary protein intake, would be sufficient to fully overcome this anabolic resistance. Importantly, these findings do not indicate that aerobic exercise is detrimental to amino acid-mediated stimulation of muscle protein synthesis in obesity. Rather, they suggest that the immediate post-aerobic exercise period does not represent the optimal temporal window for effectively eliciting amino acid-stimulated muscle protein synthesis in this population. As such, our findings have important implications for precision-based exercise and nutritional strategies, as aerobic exercise remains widely recommended for individuals with obesity due to its well-established benefits on energy balance and cardiometabolic health. Accordingly, interventions aimed at supporting skeletal muscle anabolism in obesity may need to consider the timing of amino acid or protein provision relative to aerobic exercise, as well as the recovery state of skeletal muscle.

We acknowledge that the sample size of the present study was modest. However, the magnitude and consistency of the observed effects across participants support the robustness of the muscle protein synthesis data, the primary end-point of the study. Although we did not directly assess the molecular mechanisms underlying the observed changes in muscle protein synthesis, this does not detract from the central findings, as muscle protein synthesis represents a functionally meaningful physiological end-point. Further studies are warranted to delineate the cellular and molecular pathways contributing to these responses.

In conclusion, acute aerobic exercise in humans with obesity abolishes an amino acid-stimulated increase in mixed-muscle protein synthesis during the immediate postexercise period. Thus, our findings highlight a pronounced impairment in the metabolic flexibility of skeletal muscle protein synthesis in response to plasma amino acid availability immediately after aerobic exercise in this population. Future studies should define the temporal dynamics of postexercise anabolic sensitivity to optimize synergistic effects of aerobic exercise and plasma amino acid availability on muscle protein synthesis in humans with obesity.

## Figures and Tables

**Table 1.** Subject characteristics

	AA	EX + AA

n (F/M)	4/4	4/4
Age (years)	34.1 ± 3.0	30.6 ± 2.6
Weight (kg)	102.7 ± 5.8	113.8 ± 14.0
Height (cm)	171.1 ± 2.6	176.1 ± 2.9
BMI (kg/m^2^)	34.9 ± 1.2	36.5 ± 2.0
Body fat mass (%)	35.2 ± 2.3	41.0 ± 1.6
FM (kg)	37.7 ± 3.5	47.8 ± 4.1
FFM (kg)	66.3 ± 3.5	65.5 ± 4.7
VO_2_peak (ml·min^−1^)	2230 ± 178	2306 ± 242
VO_2_peak (ml·kg^−1^·min^−1^)	22.1 ± 2.0	22.0 ± 2.9
VO_2_peak (ml·kgFFM^−1^·min^−1^)	34.0 ± 2.6	35.1 ± 2.2
Systolic blood pressure (mmHg)	119 ± 4	121 ± 5
Diastolic blood pressure (mmHg)	77 ± 4	80 ± 4
Fasting plasma glucose (mg·dl^−1^)	101.4 ± 5.0	96.9 ± 10.3
Fasting plasma insulin (μIU·ml^−1^)	11.9 ± 2.2	14.2 ± 3.7
Matsuda-ISI	3.2 ± 0.6	3.8 ± 0.8
HOMA-IR	3.1 ± 0.6	2.9 ± 0.7
Plasma triglycerides (mg·dl^−1^)	194 ± 57	194 ± 68
Total plasma cholesterol (mg·dl^−1^)	181 ± 13	1888 ± 16
Plasma HDL cholesterol (mg·dl^−1^)	40 ± 3	44 ± 2
Plasma LDL cholesterol (mg·dl^−1^)	100 ± 9.1	103 ± 10

Values are mean ± SEM. AA, amino acid infusion only; EX + AA, exercise plus amino acid infusion; BMI, body mass index; FM, fat mass; FFM, fat-free mass; VO_2_peak, peak oxygen uptake; Matsuda-ISI, Matsuda-insulin sensitivity index; HOMA-IR, homeostatic model assessment of insulin resistance; HDL, high-density lipoprotein; LDL, low-density lipoprotein; No statistically significant differences between groups were observed for any measured variables.

**Table 2.** Plasma amino acid concentrations during the Basal and Amino Acid Infusion periods, and corresponding changes from Basal

	Basal	Amino Acid Infusion	Change (*Δ*)

Alanine			
AA	311 ± 37	628 ± 53****	287 ± 21
EX + AA	335 ± 30	543 ± 29****	187 ± 17^††^
Arginine			
AA	59 ± 5	197 ± 4****	139 ± 5
EX + AA	76 ± 11	277 ± 16****	202 ± 9^††††^
Asparagine			
AA	27 ± 2	24 ± 1	−3 ± 3
EX + AA	32 ± 2	24 ± 1*	−7 ± 3
Glutamic Acid			
AA	47 ± 6	95 ± 12**	50 ± 8
EX + AA	53 ± 6	125 ± 14****	72 ± 12
Glycine			
AA	290 ± 22	564 ± 27****	262 ± 13
EX + AA	310 ± 37	566 ± 40****	256 ± 21
Histidine			
AA	68 ± 6	118 ± 6***	50 ± 12
EX + AA	77 ± 8	122 ± 6***	46 ± 7
Isoleucine			
AA	66 ± 4	202 ± 7****	138 ± 7
EX + AA	69 ± 7	208 ± 9****	140 ± 3
Leucine			
AA	137 ± 8	424 ± 16****	294 ± 16
EX + AA	139 ± 9	213 ± 14****	74 ± 7^††††^
Methionine			
AA	46 ± 1	398 ± 21****	352 ± 23
EX + AA	56 ± 6	317 ± 23****	261 ± 19^††^
Phenylalanine			
AA	62 ± 2	258 ± 7****	199 ± 6
EX + AA	71 ± 5	184 ± 10****	113 ± 5^††††^
Serine			
AA	93 ± 2	177 ± 4****	81 ± 5
EX + AA	104 ± 7	170 ± 8****	66 ± 4^†^
Threonine			
AA	125 ± 14	240 ± 16***	111 ± 6
EX + AA	144 ± 25	300 ± 31****	156 ± 15^†^
Tyrosine			
AA	75 ± 1	79 ± 1	5 ± 0
EX + AA	83 ± 11	89 ± 11	6 ± 8
Valine			
AA	226 ± 7	553 ± 30****	332 ± 29
EX + AA	245 ± 20	462 ± 22****	217 ± 17^††^
Total Amino Acids			
AA	1634 ± 55	3957 ± 131****	2297 ± 108
EX + AA	1814 ± 117	3598 ± 152****	1788 ± 86^††^
Essential Amino Acids			
AA	731 ± 13	2193 ± 73****	1476 ± 79
EX + AA	801 ± 62	1807 ± 82****	1005 ± 42^†††^
Branched Chain Amino Acids			
AA	430 ± 14	1179 ± 47****	765 ± 49
EX + AA	453 ± 30	883 ± 36****	430 ± 23^††††^
Non-essential Amino Acids			
AA	903 ± 55	1764 ± 76****	821 ± 35
EX + AA	1013 ± 62	1791 ± 78****	783 ± 47

Values are in μmol·L^−1^; Data are mean ± SEM; AA, amino acid infusion only; EX + AA, exercise plus amino acid infusion

**** *P* < 0.0001

*** *P* < 0.001

** *P* < 0.01

* *P* < 0.05 versus Basal

†††† *P* < 0.0001

††† *P* < 0.001

†† *P* < 0.01

†† *P* < 0.01 versus AA.

**Table 3.** Plasma glucose and insulin concentrations during the Basal and Amino Acid Infusion periods, and corresponding changes from Basal

	Basal	Amino Acid Infusion	Change (*Δ*)

Glucose			
AA	93.1 ± 3.9	96.7 ± 3.4	3.6 ± 2.9
EX + AA	84.3 ± 2.6	84.9 ± 2.2	0.6 ± 1.5
Insulin			
AA	18.7 ± 3.4	48.3 ± 4.7****	29.7 ± 5.5
EX + AA	19.9 ± 3.2	39.4 ± 6.5***	19.5 ± 4.7

Values are in mg·dL^−1^ (glucose) and μIU·mL^−1^ (insulin); Data are mean ± SEM; AA, amino acid infusion only; EX + AA, exercise plus amino acid infusion

**** *P* < 0.0001

** *P* < 0.01 versus Basal.
